# Peritoneopericardial diaphragmatic hernia in a healthy adult feline (*Felis catus domesticus*): diagnosis to surgical treatment - Case report

**DOI:** 10.29374/2527-2179.bjvm001820

**Published:** 2021-03-23

**Authors:** Ana Carolina de Souza Campos, Lucas Rabaça dos Santos, Fernando Elisio Torres, Juliana Letícia Rossetto Marques, Caroline Lopes Martini, Stéfani Franco de Sá Menezes, Gabriela Moscatel Fevrier, Viviane Horta Gomes

**Affiliations:** 1 Veterinarian, Programa de Pós-Graduação em Medicina Veterinária (PPGMV), Departamento de Medicina e Cirurgia Veterinária (DMCV), Instituto de Veterinária (IV) Universidade Federal Rural do Rio de Janeiro (UFRRJ), Campus Seropédica, RJ, Brasil; 2 Veterinarian, Faculdade de Veterinária (FV), Universidade Federal Fluminense (UFF), Niterói, RJ, Brasil; 3 Veterinarian, DMCV, IV, UFRRJ, Campus Seropédica, RJ, Brasil; 4 Veterinarian, PPGMV, FV, UFF, Niterói, RJ, Brasil; 5 Veterinarian, DSc. Instituto de Microbiologia Paulo de Góes, Universidade Federal do Rio de Janeiro, Rio de Janeiro, RJ, Brasil; 6 Veterinarian, Univeritas, Rio de Janeiro, RJ, Brasil; 7 Veterinarian, Universidade Federal de Viçosa, Viçosa, MG, Brasil.; 8 Veterinarian, DSc. DMCV, IV, UFRRJ, Campus Seropédica, RJ, Brasil

**Keywords:** congenital anomaly, surgery, echocardiogram, anomalia congênita, cirurgia, ecocardiograma

## Abstract

Peritoneopericardial diaphragmatic hernia (PPDH) is a communication between the abdomen and the pericardial sac generated by congenital anomalies triggered during diaphragmatic and pericardial development. This report aimed to present the case of an adult, mixed-breed cat, affected by PPDH, focusing on the period from diagnosis to successful surgical correction. The patient had a capricious appetite and weight loss for about four months and started, at the end of this period, a state of apathy. On abdominal ultrasound, the gallbladder (GB) was close to the heart, suggesting diaphragmatic discontinuity. On thoracic radiography, there were changes suggestive of PPDH, pericardial efusion or cardiomegaly with probable dilated cardiomyopathy. Based on these findings, an echocardiogram was performed, highlighting the hepatic lobe and GB internally to the pericardium, causing cardiac compression, although without severe cardiac changes. During surgery, a diaphragmatic defect of 4 cm in diameter was observed with the congested right medial hepatic lobe and hyperemic GB in the pericardial sac. The defect was sutured using the sultan pattern in separate stitches and polyamide threads. The feline returned to feeding with greater interest soon after the surgery, and after 15 days it was fed with dry food and had normal behavior. PPDH can be diagnosed in healthy adult cats, even if there are no apparent respiratory, gastrointestinal, or cardiac signs. The echocardiogram is relevant in the definitive diagnosis, in addition to excluding differential diagnoses, and simple surgical treatment with polyamide thread and sultan suture is successful.

## Resumo

A hérnia periotônio-pericárdica diafragmática (HPPD) comunica o abdome e o saco pericárdico, é gerada por anomalias congênitas deflagradas no desenvolvimento diafragmático e pericárdico. O objetivo deste relato é apresentar o caso de uma gata adulta, mestiça, acometida por HPPD, destacando do diagnóstico à correção cirúrgica bem sucedida. A paciente exibia apetite caprichoso e emagrecimento há cerca de 4 meses, iniciando ao final deste período, apatia. Na ultrassonografia abdominal a vesícula biliar (VB) estava próxima ao coração sugerindo ruptura diafragmática. Na radiografia torácica evidenciou-se alterações sugestivas de HPPD ou cardiomegalia com provável miocardiopatia dilatada. Devido tais achados realizou-se ecocardiograma destacando o lobo hepático e a VB no pericárdio comprimindo o coração sem comprometer sua função. Na cirurgia observou-se defeito diafragmático (4 cm), passagem do lobo hepático medial direito e da VB para o pericárdio. Suturou-se o defeito com padrão sultan e fio poliamida 3.0. A felina voltou a se alimentar com maior interesse logo após a cirurgia. A HPPD pode ser diagnosticada em felinos adultos saudáveis, mesmo que não haja sinais respiratórios, gastrointestinais ou cardíacos aparentes. O ecocardiograma é relevante no diagnóstico definitivo, além de excluir diagnósticos diferenciais, sendo o simples tratamento cirúrgico com fio poliamida e sutura sultan bem sucedido.

## Introduction

Peritoneopericardial diaphragmatic hernia (PPDH) is the most common congenital anomaly of the diaphragm and pericardium in feline species (Johnson, 1993). This is the result of defects during the development of these anatomical structures (Pinto Filho, 2017), generating communication between the abdomen and the pericardial sac (MacPhail, 2014). It usually presents in young cats under one year of age (Barrett & Kittrell, 1966). Persian and Maine Coon cats are more frequently affected (Banz & Gottfried, 2010; Hosgood, 1996).

The thoracic content is usually variable, being possible to visualize the liver, small intestine, spleen, omentum, pancreas, colon, uterus (Pinto Filho, 2017), and rarely the stomach (Evans & Biery, 1980). Changes in the respiratory and gastrointestinal systems can be observed (Fossum, 2014; Thomas, 1989), and cardiac changes are also reported (Hunt & Johnson, 2007; Szabo & Fischetti, 2014). Asymptomatic cases are possible when there is no involvement of the herniated viscera (Cunha et al., 2000) and, for this reason, may not be diagnosed for many years (Barrett & Kittrell, 1966; Evans & Biery, 1980).

The diagnosis is made by means of radiography or ultrasound, and sometimes both are necessary (MacPhail, 2014). Contrast radiography is used in situations where the exams mentioned have not been able to definitively diagnose PPDH (MacPhail, 2014). Surgery is the definitive treatment of hernias, and in some situations, in addition to being necessary, it is an emergency (Scheffers & Atallah, 2018). Conservative treatment may be indicated in certain patients (Szabo & Fischetti, 2014).

The prognosis for cats with PPDH with surgical correction is excellent for the return to normal function (Banz & Gottfried, 2010), becoming reserved in patients with concomitant cardiac abnormalities (MacPhail, 2014).

This report describes the diagnostic approach to surgical treatment of an adult cat with nonspecific clinical signs affected by PPDH.

## Case report

A 4-year-old mixed-breed female feline weighing 3.4 kg and negative Feline Immunodeficiency Virus/Feline Leukemia Virus tests with a history of reduced appetite and weight loss in the last 4 months was attended to. The feline had a low body condition score, apathy, mild dehydration, abdominal palpation without pain and notable changes, during cardiopulmonary auscultation no muffling or other changes were observed, refused dry food, feeding only wet cat food, and homemade food. Considering the patient’s history and clinical signs, laboratory and imaging tests were requested.

**Figure 1:**
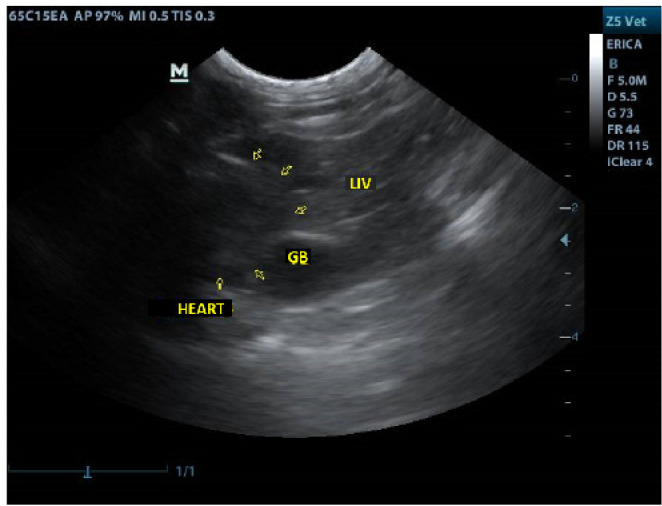
Ultrasound image of a feline, mixed-breed, 4 years old, affected by peritoneopericardial diaphragmatic hernia (cross section, right intercostal window). Notice the presence of a portion of the liver (LIV) and gallbladder (GB) in contact with the heart (arrows) in the thoracic region. Cat para Gatos - RJ (04/2020).

**Figure 2:**
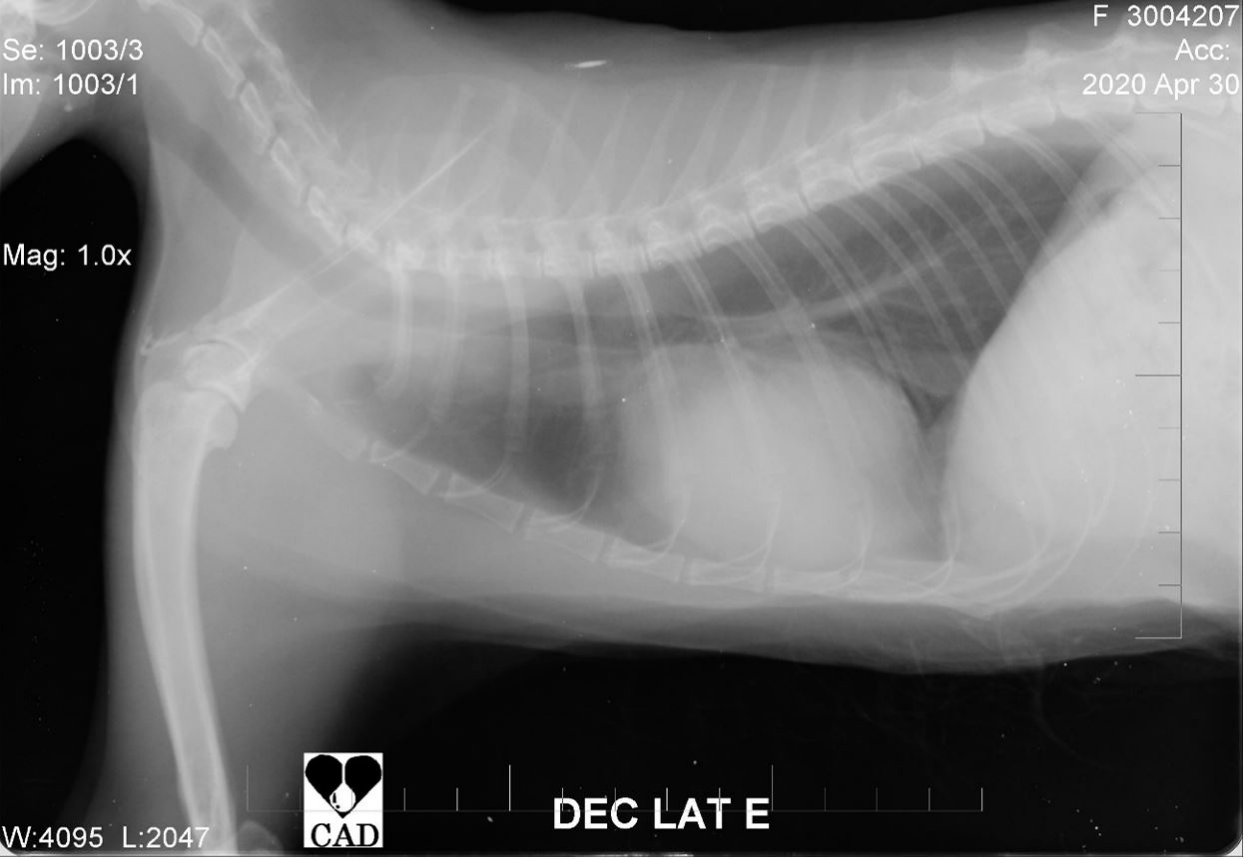
Thoracic radiography image (lateral position) of a feline, mixed-breed, 4 years old, affected by peritoneopericardial diaphragmatic hernia. Notice an increase in all cardiac chambers. Veterinary Support and Diagnosis Center - RJ (04/2020).

**Figure 3:**
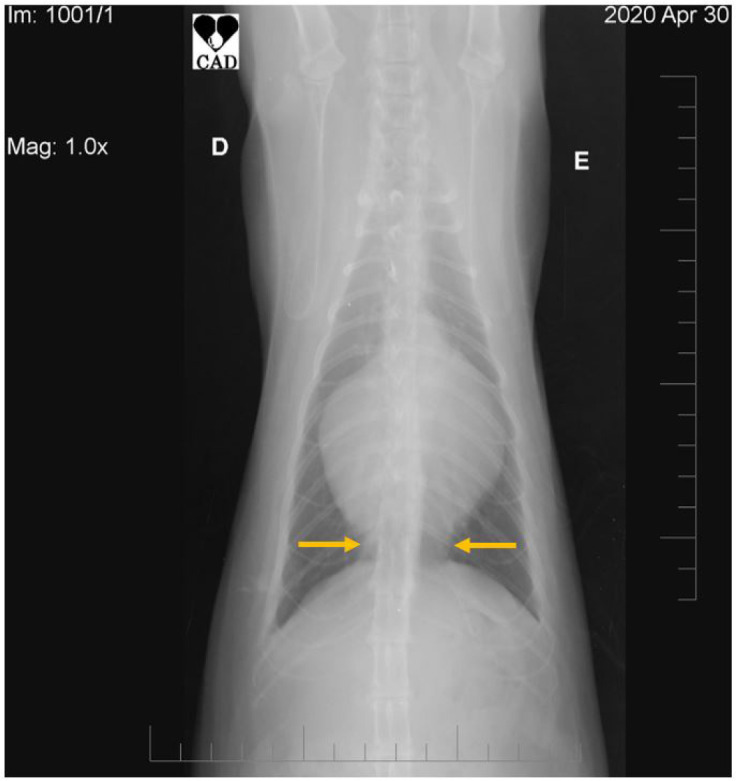
Thoracic radiography image (ventrodorsal position) of a feline, mixed-breed, 4 years old, affected by peritoneopericardial diaphragmatic hernia. Notice an increase in liquid and soft tissue density in the region of cardiodiaphragmatic contact (arrows) and an overall cardiac increase. Veterinary Support and Diagnosis Center - RJ (04/2020).

**Figure 4:**
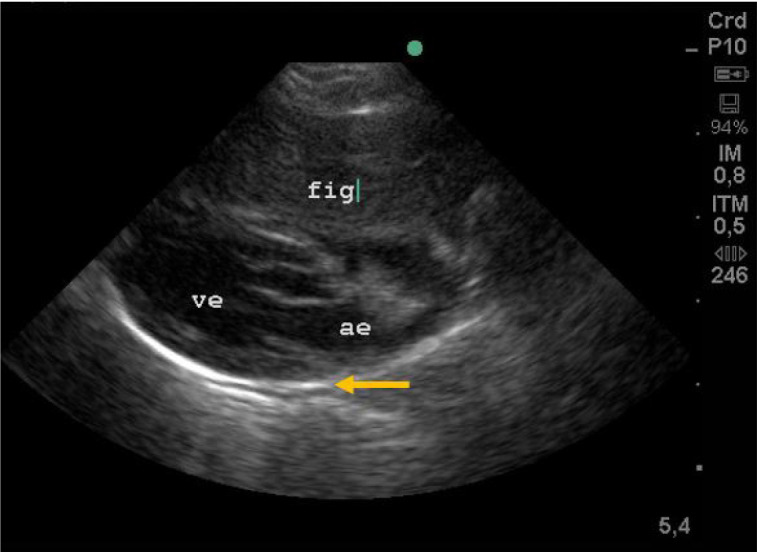
Echocardiographic image of feline, mixed-breed, 4 years old, affected by peritoneopericardial diaphragmatic hernia (left caudal parasternal window in longitudinal section of the ventricle and left atrium in the region of the left ventricular outflow tract). Notice the liver (FIG) in proximity to the left ventricle (VE) and left atrium (AE). On the arrow, evidence of the pericardial sac. Cat para Gatos RJ (05/2020).

Only a slight increase in alanine aminotransferase was observed in laboratory tests (complete blood count and biochemistry). Abdominal ultrasonography revealed the gallbladder close to the heart (Figure 1), distended by bile, with a regular wall and anechoic content. Thoracic radiography showed an overall increase of the cardiac silhouette (Figure 2), loss of cardiac waist, associated with an increase in liquid density/soft tissue in the region of cardiac diaphragmatic contact (Figure 3). Echocardiography revealed that the medial hepatic lobe and the gallbladder were inside the pericardium (Figure 4); no major cardiac changes were observed. From the results of the imaging examinations, a PPDH was diagnosed, and surgical correction was indicated.

The cat received 0.3 mg/kg of methadone (Mytedom^®^; Cristália Produtos Químicos e Farmacêuticos Ltda, SP, Brazil) intramuscularly. Twenty-five minutes after premedication, anesthesia was induced intravenously (IV) with 3.0 mg/kg propofol (Propovan^®^; Cristália Produtos Químicos e Farmacêuticos Ltda, SP, Brazil) combined with 0.2 mg/kg midazolam (Dormire^®^; Cristália Produtos Químicos e Farmacêuticos Ltda, SP, Brazil). Endotracheal intubation was performed, and anesthesia was maintained with isoflurane (Isoforine^®^; Cristália Produtos Químicos e Farmacêuticos Ltda, SP, Brazil) delivered in 100% oxygen. The dose of 0.5 mg/kg of dexamethasone was administered IV. The cat was positioned supine on a thermal blanket (Mattress Thermal Veterinary; Estek Ltda., SP, Brazil), and trichotomy and antisepsis of the surgical site were performed.

**Figure 5:**
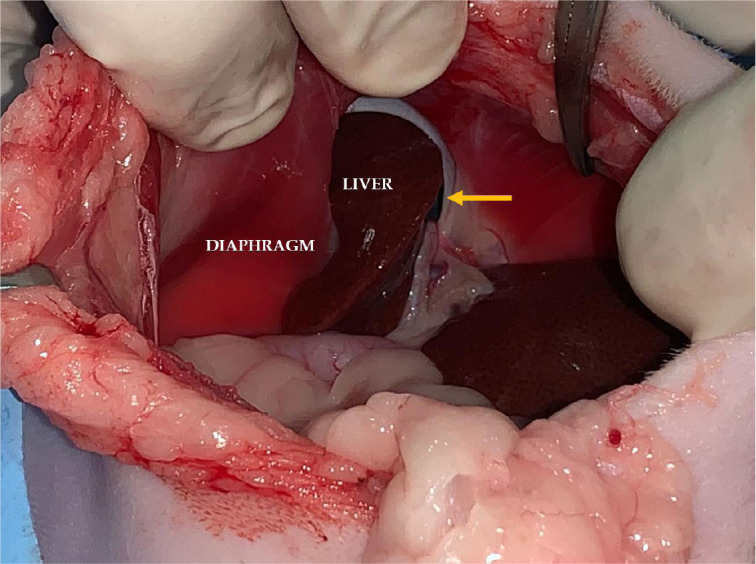
Photograph of the diaphragmatic region visualized via abdominal access after median longitudinal celiotomy of a feline, mixed-breed, 4 years old, affected by peritoneopericardial diaphragmatic hernia. Notice part of the liver inserted in the diaphragmatic defect (arrow). Cat para Gatos - RJ (05/2020).

**Figure 6:**
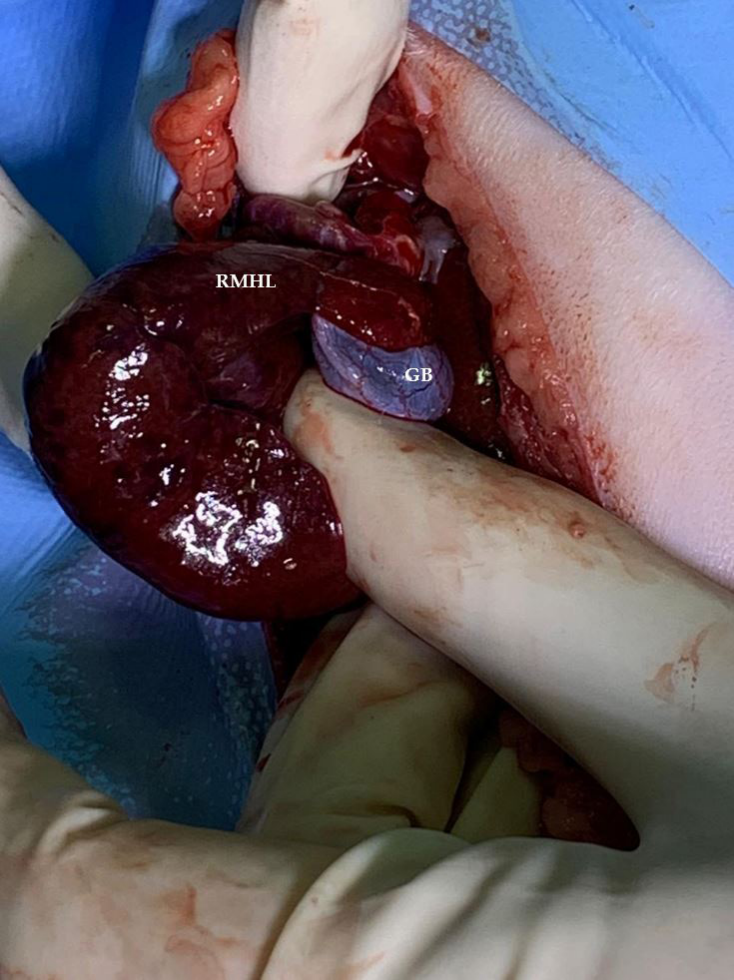
Photograph of the right medial hepatic lobe (RMHL) and the feline gallbladder (GB), mixed-breed, 4 years old, affected by peritoneopericardial diaphragmatic hernia, visualized after median longitudinal celiotomy. The moment immediately after caudal mobilization and removal of these organs from the pericardial sac. Notice the liver congestion (heterogeneous appearance), as well as dilation of the gallbladder vessels. Cat para Gatos - RJ (05/2020).

**Figure 7:**
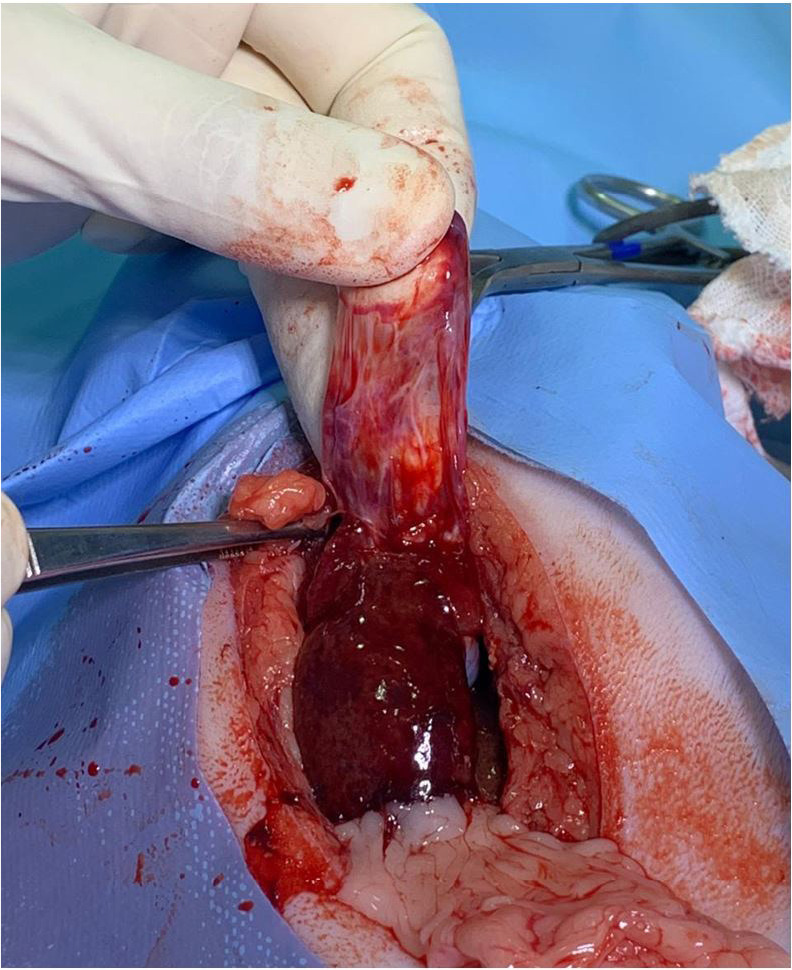
Photograph of the feline right medial hepatic lobe, mixed-breed, 4 years old, affected by peritoneopericardial diaphragmatic hernia, visualized after median longitudinal celiotomy after abdominal repositioning of this organ. The surgeon indicates the pericardial sac with a dissecting forceps. Notice the hepatic congestion (heterogeneous surface appearance). Cat para Gatos - RJ (05/2020).

Surgical access was performed by median longitudinal celiotomy in the pre-umbilical region from the xiphoid process to the retro-umbilical area. After entering the abdominal cavity, a defect of 4 cm in diameter was seen in the costal muscle portion of the diaphragm (Figure 5). From the hernia, using Metzembaum scissors, a 1 cm incision was made in the diaphragm, allowing the mobilization of the contents of the pericardial sac (right medial hepatic lobe and gallbladder). Congestion of the hepatic lobe and hyperemia of the gallbladder were observed (Figures 6 and 7).

**Figure 8:**
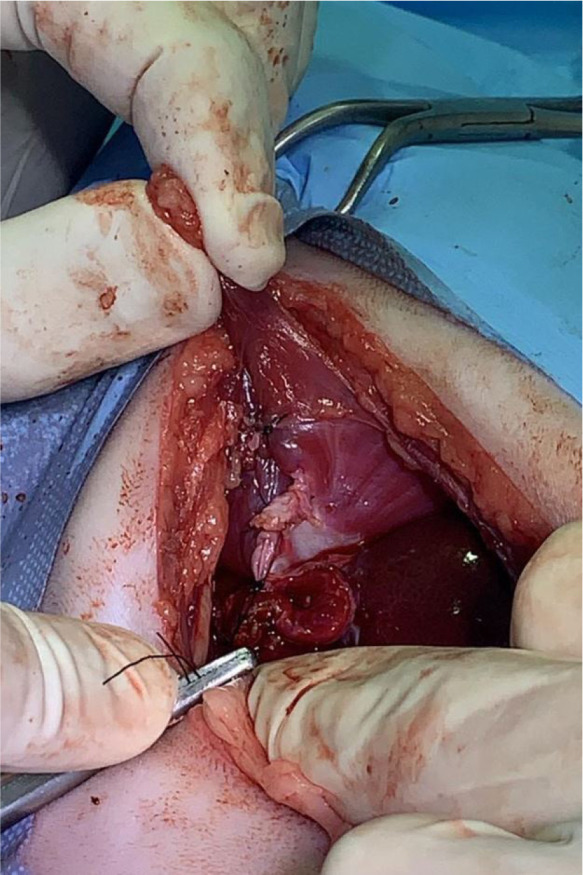
Photograph of defect raffia in the diaphragma visualized after median longitudinal celiotomy of feline, mixed-breed, 4 years old, affected by peritoneopericardial diaphragmatic hernia. Notice the polyamide thread (number 3.0) being pulled by a needle holder. Cat para Gatos - RJ (05/2020).

Then, a small incision was made in the pericardium to remove its adhesion to the diaphragm. The diaphragmatic musculature was sutured with a sultan pattern and a 3-0 polyamide thread (Figure 8) in the direction of the central tendon towards the xiphoid process. When performing the last two points, the anesthetist was asked to inflate the lung as much as possible to recover negative intrathoracic pressure. Abdominal synthesis was performed as usual. During the intraoperative period, two boluses of fentanyl (2 mcg/kg IV) were performed for pain control.

The cat was hospitalized for 24 h after the surgical procedure. Returned to eating right after the surgery, with greater interest and eating more volume than usual. The animal was discharged; meloxicam (0.1 mg/kg, once daily, for 3 days), gabapentin (10 mg/kg, twice daily, for 15 days), and tramadol hydrochloride (2 mg/kg once a day, for 5 days) were prescribed. Twenty-one days of rest were indicated. Fifteen days postoperatively, the surgical skin stitches were removed and the cat was already eating only dry food with full interest. Two months after the surgery, the animal gained weight and already showed normal behavior.

## Discussion

The peritoneal cavity of the feline is not directly juxtaposed to the pericardial cavity; the two are connected only by the caudal mediastinal pleura, which renders traumatic PPDH rare in animals (Hunt & Johnson, 2007). It is believed that the PPDH in the present patient was congenital, as it was diagnosed accidentally, with no history of trauma.

The cat did not show clinical signs associated with the presence of PPDH, such as changes in the gastrointestinal or cardiorespiratory system (Barge et al., 2019; Barrett & Kittrell, 1966; Liptak et al., 2002), being observed only in apathy, weight loss, and lack of appetite. As in this report, some animals can be diagnosed as middle-aged or older (MacPhail, 2014), may be asymptomatic, and may not even reveal pathological changes in herniated or thoracic organs (Cunha et al., 2000; Hunt & Johnson, 2007).

Hematological examinations in cats affected by PPDH may present subtle changes (Barge et al., 2019) or may not show changes (Banz & Gottfried, 2010; Liptak et al., 2002). Therefore, even in the presence of a blood cell count and serum chemistry profile within normal range or with subtle changes, as in this report, it is possible to have PPDH.

In this report, echocardiography was essential to eliminate possible differential diagnoses, such as dilated cardiomyopathy (Barrett & Kittrell, 1966; MacPhail, 2014) or pericardial effusion. The cardiac changes observed by the echocardiogram were considered mild, with no impairment of the cat’s cardiovascular function. However, patients with PPDH may have severe cardiac changes; in these cases, surgical correction is not indicated, and conservative treatment should be adopted (Szabo & Fischetti, 2014).

We opted for access by median longitudinal celiotomy because the PPDH was located in the ventral midline of the diaphragm, easily visualized using this access. In addition, accessing the linea alba allows better exposure to the sternum and abdominal wall, which can present associated birth defects (Hunt & Johnson, 2007). There is no need for associated sternotomy as has been reported (Cunha et al., 2000; Liptak et al., 2002).

During the surgical correction of the PPDH, the cranial extension of the incision, in addition to facilitating the transection of possible adhesions, also allows a wide view of both cavities, allowing the movement of the abdominal organs found in the pericardial sac without damaging them (Cunha et al., 2000). In this report, extending the defect in the diaphragm was useful for visualizing the anatomical structures, removing the adhesions between the cavities, allowing unrestricted access to the initial defect and suturing it comfortably.

The suture of the defect with polyamide thread in a sultan pattern initially, in the dorsal direction towards the ventral, as suggested by Hunt & Johnson (2007) proved to be adequate. The pericardium was separated from the peritoneum at the edges of the diaphragmatic rupture, as suggested by Barrett & Kittrell (1966). This maneuver allowed the suture of the defect without reducing the total size of the pericardium by suturing it in association with the diaphragm.

The patient remains under follow-up without showing signs of recurrence, despite the reserved prognosis in patients with PPDH who have associated cardiac changes (MacPhail, 2014), and the postoperative mortality rate in cats being 12.5% (Banz & Gottfried, 2010).

## Conclusion

Peritoneopericardial diaphragmatic hernia (PPDH) can be diagnosed in healthy adult cats, even if there are no apparent respiratory, gastrointestinal, or cardiac signs. The echocardiogram is relevant in the diagnosis and exclusion of possible differential diagnoses. The surgical treatment, through the median longitudinal celiotomy approach, using polyamide thread and sultan suture, was successful in this report.

## Availability of complementary results

For more information about the case, such as results in details of the complementary exams performed and access to more pictures of the surgical procedure or the patient’s anesthetic record, contact the authors.

The study was carried out at Clinic Cat para Gatos, Rio de Janeiro, RJ, Brasil.
